# Transcriptome sequencing and comparative analysis of cucumber flowers with different sex types

**DOI:** 10.1186/1471-2164-11-384

**Published:** 2010-06-17

**Authors:** Shaogui Guo, Yi Zheng, Je-Gun Joung, Shiqiang Liu, Zhonghua Zhang, Oswald R Crasta, Bruno W Sobral, Yong Xu, Sanwen Huang, Zhangjun Fei

**Affiliations:** 1 National Engineering Research Center for Vegetables, Beijing 100097, China; 2 Boyce Thompson Institute, Cornell University, Ithaca, NY 14853, USA; 3 Department of Ornamental Horticulture, China Agricultural University, Beijing 100094, China; 4 Key Laboratory of Horticultural Crops Genetic Improvement of Ministry of Agriculture, Sino-Dutch Joint Lab of Horticultural Genomics Technology, Institute of Vegetables and Flowers, Chinese Academy of Agricultural Sciences, Beijing 100081, China; 5 Virginia Bioinformatics Institute, Virginia Tech University, Blacksburg, VA 24061, USA; 6 USDA Robert W. Holley Center for Agriculture and Health, Tower Road, Ithaca, NY 14853, USA

## Abstract

**Background:**

Cucumber, *Cucumis sativus *L., is an economically and nutritionally important crop of the *Cucurbitaceae *family and has long served as a primary model system for sex determination studies. Recently, the sequencing of its whole genome has been completed. However, transcriptome information of this species is still scarce, with a total of around 8,000 Expressed Sequence Tag (EST) and mRNA sequences currently available in GenBank. In order to gain more insights into molecular mechanisms of plant sex determination and provide the community a functional genomics resource that will facilitate cucurbit research and breeding, we performed transcriptome sequencing of cucumber flower buds of two near-isogenic lines, WI1983G, a gynoecious plant which bears only pistillate flowers, and WI1983H, a hermaphroditic plant which bears only bisexual flowers.

**Result:**

Using Roche-454 massive parallel pyrosequencing technology, we generated a total of 353,941 high quality EST sequences with an average length of 175bp, among which 188,255 were from gynoecious flowers and 165,686 from hermaphroditic flowers. These EST sequences, together with ~5,600 high quality cucumber EST and mRNA sequences available in GenBank, were clustered and assembled into 81,401 unigenes, of which 28,452 were contigs and 52,949 were singletons. The unigenes and ESTs were further mapped to the cucumber genome and more than 500 alternative splicing events were identified in 443 cucumber genes. The unigenes were further functionally annotated by comparing their sequences to different protein and functional domain databases and assigned with Gene Ontology (GO) terms. A biochemical pathway database containing 343 predicted pathways was also created based on the annotations of the unigenes. Digital expression analysis identified ~200 differentially expressed genes between flowers of WI1983G and WI1983H and provided novel insights into molecular mechanisms of plant sex determination process. Furthermore, a set of SSR motifs and high confidence SNPs between WI1983G and WI1983H were identified from the ESTs, which provided the material basis for future genetic linkage and QTL analysis.

**Conclusion:**

A large set of EST sequences were generated from cucumber flower buds of two different sex types. Differentially expressed genes between these two different sex-type flowers, as well as putative SSR and SNP markers, were identified. These EST sequences provide valuable information to further understand molecular mechanisms of plant sex determination process and forms a rich resource for future functional genomics analysis, marker development and cucumber breeding.

## Background

Cucumber (*Cucumis sativus *L.) is an economically and nutritionally important vegetable crop cultivated world-wide and belongs to the *Cucurbitaceae *family which includes several other important vegetable crops such as melon, watermelon, squash and pumpkin. Cucumber has considerable impact on human nutrition and is among 35 fruits, vegetables, and herbs identified by the National Cancer Institute as having cancer-protective properties. Cucumber and melon have long served as the primary model systems for sex determination studies due to their diverse floral sex types [1]. Sex determination in flowering plants is a fundamental developmental process of great economical importance. Sex determination occurs by the selective arrest of either the male stamen or female carpel during development [2]. Sex expression in cucurbit species can be regulated by plant hormones and environmental factors [1]. Ethylene is highly correlated with the femaleness and has been regarded as the primary sex determination factor [3,4]. Early genetics studies indicated that there are three major sex-determining genes in cucumber and melon: *F*, *A*, and *M *[5]. Recently, the *A *gene in melon and the *M *gene in cucumber have been cloned and both encode 1-aminocyclopropane-1-carboxylic acid synthase (ACS), which is a key enzyme in ethylene biosynthesis [6,7]. In cucumber, a series of evidences strongly support that the *F *gene also encodes an ACS [8,9]. Despite such advances, the molecular mechanisms of sex expression in cucurbit species still remain largely unknown.

Cucumber is a diploid species with seven pairs of chromosomes (2n = 14). The cucumber genome is relatively small, with an estimated size of 367 Mb [10], which is similar to rice (389 Mb; [11]), and approximately three times the size of the model species *Arabidopsis thaliana *(125 Mb; [12]). Despite its economical and nutritional importance and the relatively small genome size, currently available genomic and genetic tools for cucumber are very limited. These combined with the fact that the genetic diversity of cucumber is very narrow are major factors limiting cucumber breeding. For the past 10 years, the average yields of both fresh and processing cucumbers have remained virtually unchanged in the United States [13]. Therefore, in order to develop improved crops, it is necessary to develop new resources that can be used to identify novel molecular markers that are linked to the trait of interest.

Recently the whole genome sequencing of the domestic cucumber, *C. sativus *var. *sativus *L., has been completed using a hybrid approach by combining traditional Sanger and next-generation Illumina GA sequencing technologies [14]. The completion of cucumber whole genome sequencing provides tremendous opportunities for evolutionary and comparative genomics analysis and facilitates the identification of key genes of economical and biological interests. Complementary to the whole genome sequences, Expressed Sequenced Tags (ESTs) present an alternative valuable resource for research and breeding as they provide the most comprehensive information regarding the dynamics of cucumber transcriptome. It has been reported that ESTs have played significant roles in accelerating gene discovery including gene family expansion [15,16], improving genome annotation [17], elucidating phylogenetic relationships [18], facilitating breeding programs for both plants and animals by providing SSR and SNP markers [19,20], and large-scale expression analysis [21,22]. In addition, ESTs are a robust method for rapid identification of transcripts involved in specific biological processes. Currently there are more than 64 million ESTs in the NCBI public collection, dbEST database [23]. However, only around 8,000 EST sequences are available for cucumber and approximately 150,000 for all the species in the *Cucurbitaceae *family, of which ~50,000 are in the dbEST database and ~100,000 recently generated melon ESTs are available in the Cucurbit Genomics Database [24], as compared to more than 1.5 and 2 million ESTs available for *Arabidopsis *and maize, respectively.

Recent advances in next-generation sequencing technologies allow us to generate large scale ESTs efficiently and cost-effectively. In this study, we report the generation of more than 350,000 high quality cucumber ESTs from flower buds of two near-isogenic lines, a gynoecious plant (*MMFF*) which bears only female flowers and a hermaphroditic plant (*mmFF*) which bears bisexual flowers, using Roche-454 massive parallel pyrosequencing technology. These ESTs, together with ~5,600 high quality cucumber EST and mRNA sequences available in public domains, were clustered and assembled into 81,401 unigenes, which were further aligned to cucumber genome predicted genes and annotated extensively in this study. We then performed comparative digital expression profiling analysis to systematically characterize the differences of mRNA expression levels between the two flowers with different sex types, in an attempt to identify genes playing roles in cucumber sex determination. Furthermore, putative SNP and SSR markers were identified from these ESTs.

## Results and discussion

### Cucumber EST sequence generation and assembly

**Figure 1:**
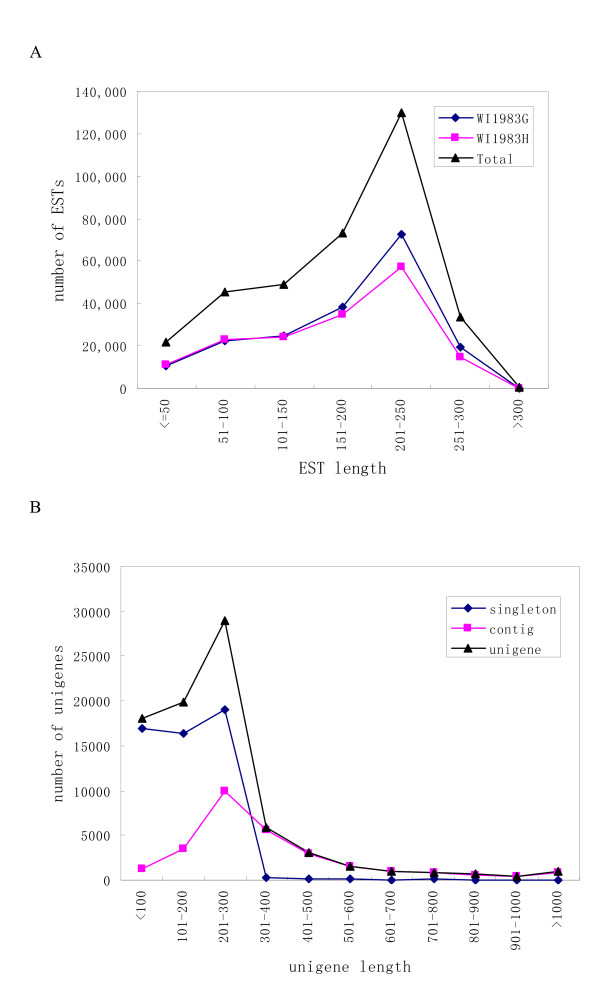
**Length distributions of cucumber ESTs (A) and assembled sequences (B)**.

We performed a half 454 GS-FLX run on each of the two flower bud samples which were collected from two near-isogenic lines, a gynoecious line (WI1983G; *MMFF*) which bears only female flowers and a hermaphroditic line (WI1983H; *mmFF*) which bears only bisexual flowers. We obtained a total of approximately 405,000 raw reads. After removing low quality regions, adaptors and all possible contaminations, we obtained a total of 353,941 high quality ESTs with an average length of 175 bp and a total length of 61.9 Mb, among which 188,255 were from WI1983G and 165,686 from WI1983H (Table 1). The length distribution of these high quality ESTs is shown in Figure 1A. Despite a significant number of ESTs were very short (<100), more than 80% fell between 100 and 300 bp in length.

**Table 1:** Statistics of cucumber ESTs generated by the 454 GS-FLX platform

	WI1983G	WI1983H	Total
**No. of reads**	188,255	165,686	353,941
**Average read length (bp)**	178.5	170.6	174.8
**Total bases (bp)**	33,608,040	28,263,433	61,871,473
**No. of reads in contigs**	162,737	139,307	302,043
**No. of reads as singletons**	25,518	26,379	51,898

The ESTs generated in this study, together with 5,196 high quality ESTs and 420 mRNA sequences available in GenBank, were subjected to cluster and assembly analyses. A total of 81,401 unigenes were obtained, among which 28,452 were contigs and 52,949 were singletons. The unigenes had an average length of 231.5 bp and a total length of approximately 18.8 Mb (Table 2). The length distributions of singletons, contigs and unigenes, respectively, are shown in Figure 1B, revealing that more than 8,000 contigs are greater than 400 bp, while only around 400 singletons are greater than 400 bp.

**Table 2:** Statistics of cucumber unigenes

	Singleton	Contig	Unigene
**No. of sequences**	52,948	28,453	81,401
**Average read length (bp)**	157.5	369.3	231.5
**Total bases (bp)**	8,340,006	10,507,878	18,847,884
**No. of unigenes only having 454 reads**	51,987	25,642	77,629
**No. of unigenes only having GenBank sequences**	1,051	69	1,120
**No. of unigenes having both 454 reads and Genbank sequences**	0	2,652	2,652
**No. of unigenes aligned to cucumber genome predicted genes**	35,117	23,407	58,524

**Figure 2:**
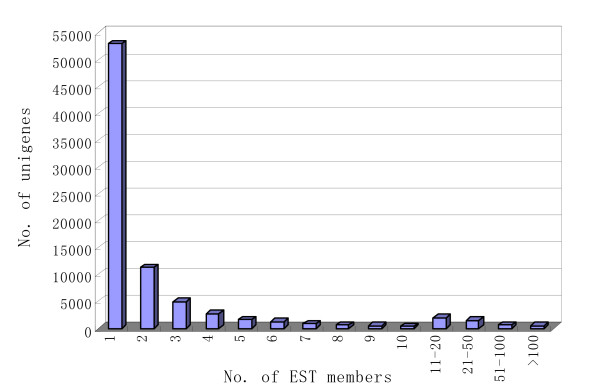
**Distribution of number of EST members in each cucumber unigene**.

The distribution of the number of ESTs in cucumber unigenes is shown in Figure 2. From our EST collection, we were able to identify a number of highly abundant transcripts in cucumber flowers. Around 4,400 transcripts have more than 10 EST members and these 4,400 transcripts (~5% of all the unigenes) contain ~62% of the EST reads.

### Alternative Splicing in Cucumber

Alternative splicing (AS) is an important regulatory mechanism in higher organisms and plays a major role in the generation of proteomic and functional diversities [25]. In plants, a wide range of processes including development, stress response and disease resistance are regulated by AS [26-28]. Currently AS of several model plant organisms including Arabidopsis and rice has been characterized at the genome scale [29,30] while AS in cucumber has not yet been investigated.

To identify AS events in cucumber genome, we mapped all cucumber ESTs to the genome predicted gene regions. We were able to identify a total of 25,917 unique intron-exon junction sites in 8,355 genes. Among these junction sites, 20,692 (80%) were consistent with those predicted from cucumber genome. A total of 530 AS events were identified in 443 cucumber genes based on the junction sites derived from EST-genome alignments (Additional file 1). These AS events were further classified into five different types: alternative 5' splice site (AltD), alternative 3' splice site (AltA), alternative position (AltP), intron retention (IntronR) and exon skipping (ExonS). Intron retention is the most prevalent AS type, comprising 55.7% of all AS events and 54.4% of all alternatively spliced genes identified in cucumber (Table 3). This is consistent with previous reports in Arabidopsis and Rice [30,31]. The relatively small number of genes were identified to have AS events in this study is probably due to the limited number of ESTs and the short length of 454 sequences, most of which were aligned entirely to single exons and did not cover the intron-exon junction sites. More RNA-seq data, especially those from different tissues and conditions, are required in order to obtain a more complete picture of alternative splicing in cucumber. The alignments of ESTs on the cucumber genome can be viewed on the cucumber genome browser in the Cucurbit Genomics Database [24].

**Table 3:** AS events and alternatively spliced genes in cucumber

AS type	Gene (%)	Event (%)
**AltD (Alternative donor site)**	63 (13.2%)	64 (12.1%)
**AltA (Alternative acceptor site)**	115 (24.1%)	118 (22.3%)
**AltP (Alternative position)**	12 (2.5%)	25 (4.7%)
**IntornR (Intron retention)**	260 (54.4%)	295 (55.7%)
**ExonS (Exon skipping)**	28 (5.9%)	28 (4.7%)

### Mapping unigenes to cucumber genome predicted genes

We further aligned cucumber unigenes to cucumber genome predicted genes. Around 72% (58,524) unigenes could be mapped, allowing 95% sequence identity and 80% length coverage (Table 2). The unmappable unigenes (22,877; 28%) in cucumber might include non-coding RNAs, fusion transcripts, relatively short and low quality singletons, UTR sequences far from the translation start or stop sites (>1000 bp), and those having incomplete coverage by the genome. It has been reported that even in Arabidopsis around 13% of the 454 ESTs can't be aligned to the predicted genes [32] and in human only 64% of the 454 reads can be mapped to the RefSeq database of well annotated human genes [33]. All the mapping results were provided in the Cucurbit Genomics Database [24]

Out of 26,682 genes predicted from the cucumber genome [14], approximately 64% (17,087) were represented by this EST collection. In addition, based on the transcript assembly described above, we found that cucumber ESTs generated in this study covered ~70% (2,625/3,772, Table 2) of genes derived from GenBank ESTs and mRNAs which were generated from various different tissues including flower, fruit and leaf. Furthermore, we compared the Arabidopsis protein sequences against cucumber unigenes using the blast program with an e-value cutoff of 1e-10 and found that ~67% of all the Arabidopsis protein sequences had at least one matching cucumber unigene. Microarray analysis in Arabidopsis indicates that 55-67% genes are expressed in a single sample [34] and studies in human and mouse also indicate that around 60-70% genes are expressed in a specific tissue [35]. All the above results indicated that the ESTs generated under the present study captured the majority of genes expressed in cucumber flower buds. These ESTs represented a significant addition to the existing cucurbit genomic resources.

### Functional annotation of cucumber transcriptome

Based on the alignments of unigenes to cucumber genome predicted genes, a total of 39,964 unique genes were obtained, including 17,087 that contained cucumber genome predicted genes and 22,877 unmappable unigenes. We named these unique genes as virtual unigenes. To infer putative functions of cucumber virtual unigenes, we compared their sequences against GenBank non-redundant protein database (nr) with an e value cutoff of 1e-5. The analysis indicated that 20,023 (50.1%) virtual unigenes had significant matches in the nr database, among which 15,126 were cucumber genome predicted genes (88.5% of the 17,087 EST-matched predicted genes) and 4,897 unmappable unigenes (21.4% of all unmappable unigenes). The low percentage (21.4%) of cucumber unmappable unigenes that can be assigned a putative function might be mainly due to the short sequence reads generated by the 454 sequencing technology and the relatively short sequences of the resulting unigenes (Table 1 and 2), most of which probably lack the conserved functional domains. Another possible reason is that some of these unigenes might be non-coding RNAs.

**Figure 3:**
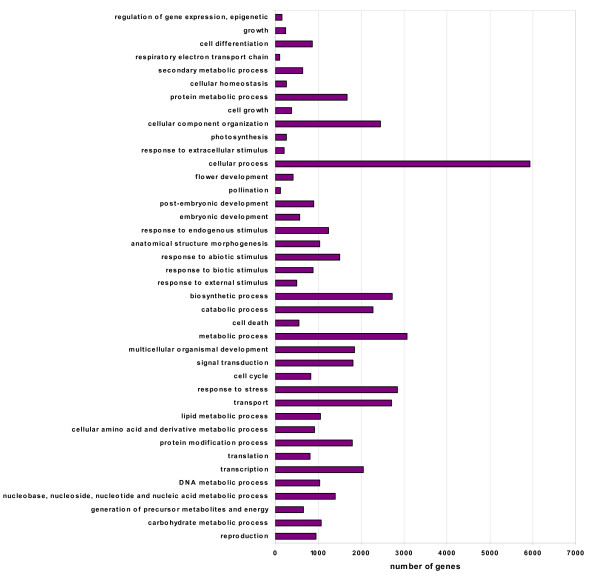
**Number of cucumber unigenes in each functional category**. Cucumber unigenes were classified into different functional groups based on a set of plant specific GO Slims in the biological process category.

Gene Ontology (GO) terms were further assigned to cucumber virtual unigenes based on their sequence similarities to known proteins in the UniProt database annotated with GO terms as well as InterPro and Pfam domains they contain. A total of 15,901 virtual unigenes (39.8%) were assigned at least one GO term, among which 13,620 were assigned at least one GO term in the biological process category, 13,799 in the molecular function category and 12,982 in the cellular component category. These virtual unigenes were further classified into different functional categories using a set of plant-specific GO slims, which are a list of high-level GO terms providing a broad overview of the ontology content [36]. Figure 3 shows the functional classification of cucumber virtual unigenes into plant specific GO slims within the biological process category. Cellular process, metabolic process, and biosynthetic process were among the most highly represented groups, indicating the flower buds were undergoing rapid growth and extensive metabolic activities. It is worth noting that GO annotations revealed 417 and 129 genes involved in flower development and the pollination process, respectively. Genes involved in other important biological processes such as stress response, signal transduction, and cell differentiation were also identified through GO annotations.

### Biochemical pathways

To further demonstrate the usefulness of cucumber ESTs generated in the present study, we identified biochemical pathways represented by the EST collection. Annotations of cucumber unigenes were fed into the Pathway Tools [37] and this process predicted a total of 343 pathways represented by a total of 5,342 unigenes, which belonged to 1,407 virtual unigenes. These predicted pathways represented the majority of plant biochemical pathways for compound biosynthesis, degradation, utilization, and assimilation, and pathways involved in the processes of detoxification and generation of precursor metabolites and energy. A database containing all the predicted cucumber pathways has been developed and is available through the Cucubit Genomics Database [24].

Enzymes catalyzing almost all steps in several major plant metabolic pathways including Calvin cycle, glycolysis, gluconeogenesis, pentose phosphate pathway, and several important secondary metabolite biosynthesis pathways including carotenoid biosynthesis and flavonoid and anthocyanin biosynthesis, could be represented by unigenes derived from the cucumber EST collection. Moreover, genes encoding oxidosqualene cyclase, an enzyme in the cucurbitacin biosynthesis pathway, were also found in the EST collection. All these evidences supported that the ESTs generated under this study provided a valuable resource for cucumber gene discovery and future functional analysis.

### Comparison of transcriptomes between gynoecious and hermaphroditic flowers

Cucumber is a model system for sex determination studies due to its diverse floral sex types [1]. During the past several years, significant progresses have been made in elucidating the mechanisms of plant sex determination, an important and fundamental developmental process of flowering plants, as exemplified by cloning several major sex-determining genes in cucurbit species [6,7,38]. Despite such advances, little is known about transcriptome dynamics of flowers with different sex types. In the present study, we systematically compared transcriptome dynamics between flowers of two isogenic lines, a gynoecious plant and a hermaphroditic plant, using a digital expression profiling approach.

Digital expression profiling, also called tag sampling or RNA-seq, has been proved to be a powerful and efficient approach for gene expression analysis at the genome level [39] and offers several advantages over microarray technologies (See review in [40]). Due to the rapid advances in next generation sequencing technologies, the digital expression profiling approach becomes more and more widely used. It has been reported that with EST collections as small as 1,000 reads, quantitative expression data for numerous moderately and highly expressed genes can be generated [21,41,42]. SAGE, which is also a tag-count based gene expression analysis technology and has been widely used for transcriptome profiling study, usually collects 50,000 to 100,000 short tags for each sample [43]. In the present study, we collected more than 160,000 tags for each of the two samples (Table 1), providing sufficient coverage to identify the majority of genes of interest.

Our digital expression profiling analysis identified a total of 214 differentially expressed genes, among which 90 showed higher expression in gynoecious flowers and 124 showed higher expression in hermaphroditic flowers (Additional file 2). Few transcription factors other than a maize DELLA protein D8 [44] and a melon zinc finger protein CmWIP1 [38] have been functionally associated with the plant sex determination process. In this study we identified five transcription factors showing significantly higher expression in gynoecious flowers and six showing significantly higher expression in hermaphroditic flowers (Additional file 2).

Recently a C2H2 zinc-finger transcription factor in melon, CmWIP1, has been cloned and expression of *CmWIP1 *leads to carpel abortion, resulting in the development of unisexual male flowers [38]. In the present study, two zinc finger transcription factors (CU23681 and CU13995) were found to have higher expression in hermaphroditic flowers. They belong to different zinc finger transcription factor families from that of CmWIP1, as CU23681 belonging to the C2C2-GATA family and CU13995 to the VOZ family.

It has been reported that auxin can induce pistillate flower formation through its stimulation of ethylene production [45]. An Aux/IAA transcription factor (CU29035) was found to have higher expression in hermaphroditic flowers. Aux/IAA genes are early auxin responsive genes and their proteins function as active repressors of secondary auxin responsive genes [46]. Lower expression of the Aux/IAA gene in gynoecious flowers could result in higher expression of secondary auxin responsive genes thus induce femaleness. Consistent with this, an auxin-induced protein (CU23408) showed higher expression in gynoecious flowers in the present study.

Brassinosteroids (BRs) can induce femaleness in cucumber and this induction could be mediated, at least in part, by brassinosteroid-induced production of ethylene [47]. In the present study, a gene (CU27987) belonging to the BZR1-BES1 family showed higher expression in hermaphroditic flowers. BZR1-BES1 family proteins represent a novel class of plant transcription factors and are key components of the BR signaling pathway [48]. In Arabidopsis, BZR1 serves as a positive regulator of the BR signaling pathway, with a role in feedback regulation of BR biosynthesis [49]. It's worth noting that two additional genes involved in BR signaling also showed higher expression in hermaphroditic flowers. One is BRI1 (CU14635), a receptor of BRs [50]. The other (CU3495) encodes a BRI1-associated receptor kinase. In Arabidopsis, the gene has been reported to interact with BRI1 and modulate BR signaling [51,52].

In Drosophila, a MYC transcription factor, daughterless (DA), provides an essential maternal component in the control of sex determination [53]. However, the role of MYC transcription factors in plant sex determination has not been documented. We found that a MYC transcription factor (CU12949) showed higher expression in hermaphroditic flowers.

Other putative transcription factors identified in this study, such as BEL1-like homeodomain protein, bHLH protein, WRKY DNA-binding protein, and NAC domain protein, have been found to regulate various processes of plant development, while a relationship between these transcription factors and plant sex determination has not been previously documented. In addition, among the genes differentially expressed in the two different sex-type flowers are several protein kinases. The correlation of transcription factors and protein kinases with sex determination suggested a pool of putative regulatory elements for future functional analysis. Furthermore, a large number of genes that have not associated with plant sex determination before were differentially expressed, suggesting additional pool of genes for further analysis.

### Over-represented biological processes in differentially expressed genes

We further identified GO terms in the biological process category that were over-represented in the lists of genes showing higher expression in gynoecious and hermaphroditic flowers, respectively (Table 4 and 5). These GO terms serve as indications of significantly different biological processes undergoing in flowers of the two different genotypes. GO terms including biopolymer metabolic process, cellular biopolymer metabolic process, cellular macromolecule metabolic process, macromolecule metabolic process, and primary metabolic process, were enriched in both lists of genes, indicating that same biological processes could require different sets of genes during gynoecious and hermaphroditic flower development to maintain their activities. However, striking differences were found between these two lists of enriched GO terms. It is worth noting that GO terms related to responses to different kinds of abiotic/biotic stresses were highly enriched in genes showing higher expression in gynoecious flowers. It has been reported that a number of environment variables, such as light, temperature, water stress, and disease, as well as exogenous treatment of hormones or other growth-regulating substances, can directly influence plant sex expression [54,55]. Factors including low temperature, low levels of light intensity, short-day treatment, low levels of carbon monoxide in the atmosphere, and exogenous application of auxins can promote cucumber female and depress male sex expression [54]. The results obtained from the present study could provide molecular cues underlying the effects of environmental factors on cucumber sex expression. Differences of other enriched GO terms included translation and system development that were enriched in genes showing higher expression in gynoecious flowers, and proteolysis and chromatin and chromosome organization that were enriched in genes showing higher expression in hermaphroditic flowers (Table 4 and 5). However, further studies are required to determine whether these biological processes are related to flower sex determinations.

**Table 4:** GO terms within the biological process category significantly enriched in genes showing higher expression in gynoecious flowers

GO term ID	description	adjusted p value
GO:0008152	metabolic process	0.01664
GO:0044237	cellular metabolic process	0.01664
GO:0010467	gene expression	0.01664
GO:0009651	response to salt stress	0.01778
GO:0009409	response to cold	0.01950
GO:0006970	response to osmotic stress	0.02555
GO:0044238	primary metabolic process	0.02555
GO:0006412	translation	0.02600
GO:0034960	cellular biopolymer metabolic process	0.02948
GO:0009628	response to abiotic stimulus	0.03021
GO:0044260	cellular macromolecule metabolic process	0.03575
GO:0050896	response to stimulus	0.04116
GO:0006950	response to stress	0.04854
GO:0009266	response to temperature stimulus	0.04854
GO:0043283	biopolymer metabolic process	0.04854
GO:0031537	regulation of anthocyanin metabolic process	0.04854
GO:0045944	positive regulation of transcription from RNA polymerase II promoter	0.04854
GO:0034961	cellular biopolymer biosynthetic process	0.04854
GO:0048731	system development	0.04854
GO:0043284	biopolymer biosynthetic process	0.04854
GO:0043170	macromolecule metabolic process	0.04854
GO:0048522	positive regulation of cellular process	0.04854
GO:0048518	positive regulation of biological process	0.04854
GO:0051707	response to other organism	0.04854
GO:0042742	defense response to bacterium	0.04854

**Table 5:** GO terms within the biological process category significantly enriched in genes showing higher expression in hermaphroditic flowers

GO term ID	description	adjusted p value
GO:0006508	proteolysis	1.85E-05
GO:0030163	protein catabolic process	1.85E-05
GO:0043283	biopolymer metabolic process	4.39E-05
GO:0043170	macromolecule metabolic process	6.27E-05
GO:0043285	biopolymer catabolic process	8.11E-05
GO:0019538	protein metabolic process	0.00012
GO:0009056	catabolic process	0.00017
GO:0009057	macromolecule catabolic process	0.00017
GO:0034960	cellular biopolymer metabolic process	0.00027
GO:0044260	cellular macromolecule metabolic process	0.00045
GO:0008152	metabolic process	0.00069
GO:0044238	primary metabolic process	0.00077
GO:0050794	regulation of cellular process	0.00078
GO:0050789	regulation of biological process	0.00237
GO:0006278	RNA-dependent DNA replication	0.00237
GO:0044237	cellular metabolic process	0.00237
GO:0019941	modification-dependent protein catabolic process	0.00398
GO:0043632	modification-dependent macromolecule catabolic process	0.00424
GO:0065007	biological regulation	0.00448
GO:0051603	proteolysis involved in cellular protein catabolic process	0.00448
GO:0044257	cellular protein catabolic process	0.00458
GO:0007165	signal transduction	0.00711
GO:0006325	chromatin organization	0.00775
GO:0006333	chromatin assembly or disassembly	0.01263
GO:0007154	cell communication	0.01430
GO:0044267	cellular protein metabolic process	0.01528
GO:0034962	cellular biopolymer catabolic process	0.01528
GO:0051276	chromosome organization	0.01885
GO:0006357	regulation of transcription from RNA polymerase II promoter	0.02912
GO:0044265	cellular macromolecule catabolic process	0.03373
GO:0044248	cellular catabolic process	0.03373
GO:0007242	intracellular signaling cascade	0.03548
GO:0006260	DNA replication	0.03667
GO:0034645	cellular macromolecule biosynthetic process	0.03769
GO:0009059	macromolecule biosynthetic process	0.03769
GO:0034961	cellular biopolymer biosynthetic process	0.04996

### Identification of Simple Sequence Repeats (SSRs) and Single Nucleotide Polymorphisms (SNPs)

Both SSRs and SNPs are valuable markers for plant breeding programs. It has been reported that approximately 3-7% of expressed genes contain putative SSR motifs, mainly within the un-translated regions of the mRNA [56]. SSR markers derived from EST sequences have been extensively used in constructing genetic maps of cucurbit species [20,57]. In the present study, we performed a general screen on the cucumber unigene dataset for the presence of SSRs. A total of 3,130 SSRs were found in 2,860 unigenes, whereas only 56 SSRs were found in unigenes containing only GenBank sequences. We excluded mononucleotide SSRs in our analysis because of the common homopolymer errors found in 454 sequencing data. The major types of the identified SSRs were trinucleotide (1,556) and dinucleotide (1,413), followed by tetranucleotide (89), pentanucleotide (46) and hexanucleotide (26). The most frequent SSR motif is AAG/CTT (769), followed by AG/CT (726), AT/TA (547) and AAT/ATT (204). Of the 2,860 SSR-containing unigenes, 1,679 (59%) had sufficient flanking sequences for primer design. The complete list of SSRs and their corresponding primer pair information were provided in Additional file 3.

Since the ESTs generated under the present study using the 454 technology are from two different cultivars, we expect SNPs to be present in our EST collection. We identified a total of 114 SNPs between WI1983G and WI1983H, among which 42 were transitions, 16 were transversions, and 56 were indels (Additional file 4). The frequency of SNP occurrence in our EST collection is relatively low, which is not unexpected since the sequences were derived from two near-isogenic lines.

In summary, the SSRs and SNPs identified in this study provided a valuable resource for future studies on genetic linkage mapping and the analysis of interesting traits in cucumber.

## Conclusion

In this study, we describe the generation of more than 350,000 cucumber cDNA sequences from flower buds of two near-isogenic lines with different floral sex types, a gynoecious line and a hermaphroditic line, using the rapid and cost-effective massive parallel pyrosequencing technology. Currently in public domains, only ~8,000 ESTs are available for cucumber and ~150,000 for all the cucurbit species. The ESTs generated in the present study represent a significant addition to the existing genomics and functional genomics resources of cucurbit species. These ESTs have been used to facilitate the annotation of cucumber genome [14] and to identify alternatively spliced genes. In addition, these ESTs can also be served as a valuable source to derive SSR and SNP markers, which can help to further identify genes linked to interesting traits. A biochemical pathway database containing more than 300 predicted metabolite pathways was derived from these EST sequences. Digital expression analysis by comparing transcriptomes of two sex-type flowers provided some novel insights into the molecular mechanisms of cucumber sex determination, as well as a rich list of candidate genes for further functional analysis. To facilitate public usages of this EST resource, all the EST sequences, annotations, their alignments to the cucumber genome, and the derived pathway database have been made available in a searchable manner through the Cucurbit Genomics Database [24].

## Methods

### Plant material

Seeds of gynoecious (*Cucumis sativus *L. var *sativus *cv WI1983G; *MMFF*) and hermaphrodite (*C. sativus *L. var *sativus *cv WI1983H; *mmFF*) nearly isogenic cucumber lines were kindly provided by Dr J. E. Staub (University of Wisconsin, Madison, USA). WI1983G originated from a cross between inbred WI5821 and WI5822 [58]. An andromonoecious near-isogenic line WI1983A (*mmff*) was developed using a hermaphrodite line as the donor parent. Five direct backcrosses to WI1983G were made followed by three subsequent generations of self-pollination. The hermaphrodite WI1983H line was selected from a cross between WI1983G and WI1983A [59]. Seeds were germinated and grown in trays containing a soil mixture (peat: sand: pumice, 1:1:1, v/v/v). Plants were adequately watered and grown at day/night temperatures of 24/18°C with a 16-h photoperiod. Flower buds of approximately 5 mm in diameter, which represents a critical stage of cucumber sex determination [60], were collected from both lines and immediately frozen in liquid nitrogen. Frozen flower buds were stored at -80°C till use.

### cDNA preparation and sequencing

Total RNA was extracted from cucumber flower buds using the TRIzol Reagent (Invitrogen, USA). mRNA was purified from the total RNA using the Oligotex mRNA Midi Kit (QIAGEN, Germany). Double-strand cDNA was then synthesized using the SMART cDNA Library Construction kit (Clontech, USA) following the manufacturer's protocol. The PCR products of cDNA were purified using the QIAquick PCR Purification Kit (QIAGEN, Germany) and checked for quality using the Agilent 2100 Bioanalyzer. Approximately 10 ug cDNA from each of the two flower samples were used for sequencing on a GS-FLX platform. A half-plate sequencing run was performed for each sample at the Virginia Bioinformatics Institute Core Laboratory Facility following manufacturer's protocols. All the sequences can be downloaded and queried at the Cucubit Genomics Database [24].

### cDNA sequence processing and assembly

The raw 454 sequence files in SFF format were base called using the Pyrobayes base caller [61]. In addition, around 7,000 EST and mRNA sequences were collected from GenBank in April, 2009. All these sequences were then processed to remove low quality regions and adaptor sequences using programs LUCY [62] and SeqClean [63]. The resulting high quality sequences were then screened against the NCBI UniVec database and E. coli genome sequences, as well as cucumber ribosomal RNA and chloroplast genome sequences, to remove possible contaminations. Sequences shorter than 30 bp were discarded. The processed 454 and GenBank sequences were assembled using the iAssembler program [64], which uses MIRA [65] and CAP3 [66] as the core assembly engines. The program performs post-assembly quality checking and automatically corrected mis-assemblies. The post-assembly quality checking mainly include 1) aligning each cDNA sequence to its corresponding unigene sequence to identify mis-assemblies; and 2) comparing unigene sequences against themselves to identify sequences from same genes that were not assembled together.

### Mapping ESTs and unigenes to cucumber genome predicted genes and identification of alternatively spliced genes

Based on full length cDNA analysis in other plant species, the majority of plant genes have 5' and 3' UTRs less than 1,000 bp [67]. For each cucumber genome predicted gene, the gene region was defined as the region from up to 1,000 bp upstream of the translation start site to up to 1,000 bp downstream of the translation stop site, allowing no overlap with the neighboring genes. ESTs and unigenes were aligned to the gene regions using SPALN [68] for those longer than 100 bp and BLAT [69] for those shorter than 100 bp. Alternative splicing events and alternatively spliced genes were identified using a custom perl script based on the alignments of ESTs to the cucumber genome predicted genes.

### Cucumber gene annotation and pathway prediction

Cucumber unigenes were blasted against GenBank non-redundant protein (nr) and UniProt databases with a cutoff e value of 1e-5. The unigene sequences were also translated into proteins using ESTScan [70] and the translated protein sequences were then compared to InterPro and pfam domain databases. The gene ontology (GO) terms were assigned to each unigene based on the GO terms annotated to its corresponding homologues in the UniProt database [71], as well as those to InterPro and pfam domains using interpro2go and pfam2go mapping files provided by the GO website [72], respectively. The GO annotations of cucumber unigenes were mapped to the plant-specific GO slim ontology using the map2slim script [36] and the unigenes were classified into different functional groups based on these GO slims. The annotations of cucumber unigenes were then formatted into the PathoLogic format and used to predict cucumber biochemical pathways using the Pathway Tools [37].

### Identification of differentially expressed genes, SNPs and SSRs

Following cDNA sequence assembly and unigene mapping to cucumber genome predicted genes, transcript count information for sequences corresponding to each gene was associated with the corresponding tissue source to obtain relative expression levels following normalization to the total number of sequenced transcripts per sample. Significance of differential gene expression was determined using the R statistic described in Stekel et al. [73] and the resulting raw p values were converted to q values for multiple test corrections [74]. Genes with fold change greater than two and q value less than 0.05 were identified as differentially expressed genes. GO terms enriched in the set of differentially expressed genes were identified using GO::TermFinder [75], requiring p values adjusted for multiple testing to be less than 0.05.

SSRs were identified from the unigenes using the MISA program [76]. The minimum repeat number was six for dinucleotide and five for tri-, tetra-, penta- and hexa-nucleotide and the maximal distance interrupting two SSRs in a compound microsatellite was 100 bp. Primer pairs flanking each SSR loci were designed using the Primer3 program [77]. SNPs in the cDNA sequences between WI1983G and WI1983H were identified with PolyBayes [78]. To eliminate errors introduced by PCR amplification during the cDNA synthesis step and homopolymer errors introduced by the 454 pyrosequencing technology, and to distinguish true SNPs from allele differences, we further filtered the PolyBayes results and only kept SNPs meeting all the following criteria: 1) at least 2× coverage at the potential SNP site for each cultivar; 2) not an indel site surrounded by long stretch (> = 3) homopolyers; 3) no same bases at the potential SNP site between the two cultivars.

## Authors' contributions

SG and YZ performed the sequence analysis. JG performed the alternative splicing analysis. SL prepared cDNA samples for 454 sequencing. ZZ and YX helped with data interpretation. ORC and BWS helped with the 454 sequencing. ZF and SH designed the experiment and provided guidance on the whole study. ZF was also involved in sequence analysis and wrote the manuscript. All authors have read and approved the manuscript.

## Supplementary Material

Additional file 1 **List of alternatively spliced genes**. The table provides the list of alternative splicing events and alternatively spliced genes identified from cucumber ESTs. Click here for file

Additional file 2 **List of differentially expressed genes**. The table provides the list of genes differentially expressed in flowers of gynoecious (WI1983G) and hermaphroditic (WI1983H) plants. Click here for file

Additional file 3 **Cucumber SSRs**. The table provides the list of SSRs identified from cucumber ESTs, their motif sequences and surrounding primer pair information. Click here for file

Additional file 4 **Cucumber SNPs**. The table provides the list of SNPs identified from the cucumber EST collection. Click here for file
